# Early diagnosis of candidemia in intensive care unit patients with sepsis: a prospective comparison of (1→3)-β-D-glucan assay, *Candida *score, and colonization index

**DOI:** 10.1186/cc10507

**Published:** 2011-10-22

**Authors:** Brunella Posteraro, Gennaro De Pascale, Mario Tumbarello, Riccardo Torelli, Mariano Alberto Pennisi, Giuseppe Bello, Riccardo Maviglia, Giovanni Fadda, Maurizio Sanguinetti, Massimo Antonelli

**Affiliations:** 1 Institute of Microbiology, Università Cattolica del Sacro Cuore, Rome, Largo F. Vito 1, 00168, Italy; 2 Department of Intensive Care and Anesthesiology, Università Cattolica del Sacro Cuore, Rome, Largo F. Vito 1, 00168, Italy; 3 Institute of Infectious Diseases, Università Cattolica del Sacro Cuore, Rome, Largo F. Vito 1, 00168, Italy

**Keywords:** glucan, *Candida *score, colonization index, invasive candidiasis, diagnosis

## Abstract

**Introduction:**

The culture-independent serum (1→3)-β-D-glucan (BG) detection test may allow early diagnosis of invasive fungal disease, but its clinical usefulness needs to be firmly established. A prospective single-center observational study was conducted to compare the diagnostic value of BG assay, *Candida *score (CS), and colonization index in intensive care unit (ICU) patients at risk for *Candida *sepsis.

**Methods:**

Of 377 patients, consecutively admitted to ICU for sepsis, 95 patients having an ICU stay of more than five days were studied. Blood specimens for fungal culture and BG measurement were obtained at the onset of clinical sepsis. For CS and colonization index calculations, surveillance cultures for *Candida *growth, and/or clinical data were recorded.

**Results:**

Sixteen (16.8%) patients were diagnosed with proven invasive fungal infection, 14 with candidiasis (13 candidemia and 1 mediastinitis) and 2 with pulmonary aspergillosis or fusariosis. Of 14 invasive *Candida*-infection patients, 13 had a serum sample positive for BG, 10 had a CS value ≥3, and 7 a colonization index ≥0.5. In the 12 candidemic patients, a positive BG result was obtained 24 to 72 hrs before a culture-documented diagnosis of invasive candidiasis. The positive and negative predictive values for the BG assay were higher than those of CS and colonization index (72.2% versus 57.1% and 27.3%; and 98.7% versus 97.2% and 91.7%, respectively).

**Conclusions:**

A single-point BG assay based on a blood sample drawn at the sepsis onset, alone or in combination withCS, may guide the decision to start antifungal therapy early in patients at risk for *Candida *infection.

## Introduction

Invasive candidiasis (IC) in non-neutropenic patients admitted to the intensive care unit (ICU) is a serious problem [1-3] and, of particular concern, associated with high mortality [4], especially when prompt and adequate antifungal treatment is not administered [5-8]. Furthermore, outcome is dependent on the causative *Candida *species and management of the primary focus of infection [9,10], and continues to be suboptimal [11].

Early identification of ICU patients with signs of sepsis at high risk of IC is challenging due to the complexity of the patients' underlying conditions and low yield of fungal cultures [12]. However, the timely recognition of IC is essential to driving clinical decision processes and dictating specific therapeutic strategies [13].

Measurement of the serum (1→3)-β-D-glucan (BG) is a non-invasive testing for circulating fungal cell wall components [14], that allows the systematic screening and prompt identification of fungal infections (with the exception of cryptococcosis and zygomycosis) [15]. This test is considered an aid for the diagnosis of fungemia and deep-seated mycoses including IC [16-18], and it appears to be useful for patients with hematological malignancies [16,17]. BG rises before infection becomes clinically apparent [16,17], but the high false-positive rates [19,20] make it necessary to refine its utility as a tool for the early diagnosis of invasive fungal infections (IFIs) [15].

At present, only clinical prediction rules of the risk of IC [21-23] or simpler scoring systems that differentiate patients with *Candida *colonization from those with ongoing but still occult *Candida *infection [24,25] are available. Their discriminative role varies; for example, a *Candida *score (CS) ≥3 was shown to select non-neutropenic critically ill patients at risk for IC more accurately than the *Candida *colonization index [25]. Although based on preliminary data, León *et al. *[26] underscored the clinical usefulness of combining CS and serum levels of BG to discriminate between colonization and proven *Candida *infection.

The present study was conducted to compare the diagnostic value of CS, colonization index, and the serum BG detection in a prospective cohort of ICU patients at risk for *Candida *sepsis.

## Materials and methods

### Patients and specimen collection

**Figure 1:**
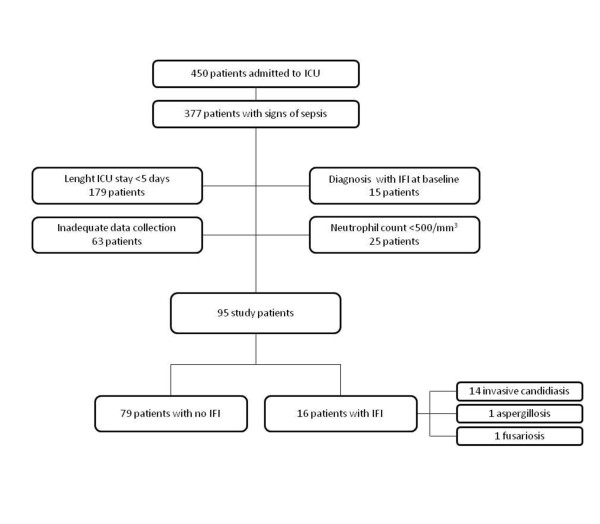
**Flow chart of the study patients**. ICU, intensive care unit; IFI, invasive fungal infection.

The study was conducted in an 18-bed adult ICU of a tertiary university teaching hospital admitting approximately 900 patients per year. The local institutional review committee approved the study, and informed consent was waived because of the observational nature of this study. All patients consecutively admitted to our ICU between March and August 2010 with signs of sepsis [27] were eligible for enrollment in this study. Patients were enrolled if they had stayed in the ICU for more than five days, had not been diagnosed with and then treated for IFI at baseline, and had a neutrophil count ≥500/mm^3 ^(Figure 1).

Age, gender, Simplified Acute Physiology Score II (SAPS II) [28] and Sequential Organ Failure Assessment (SOFA) [29], primary diagnosis, presence of various known risk factors for candidiasis (for example, an indwelling venous catheter, broad-spectrum antibiotic therapy, or treatment with corticosteroids), abdominal surgery, and outcome were recorded. Variables potentially influencing BG test results, such as renal replacement therapy, bacteremia, and recent administration of albumin, immunoglobulin products, or a β-lactam antibiotic (that is, amoxicillin-clavulanic acid or piperacillin-tazobactam) [20,30], were also recorded.

Antifungal treatment was started at the discretion of the attending physician.

For all patients, specimens from the *Candida *surveillance sites, such as rectum, oropharynx, skin (axillary surface), and urinary tract, were obtained on the day of admission to the ICU and at Days 0 and 3 following enrollment, and once a week thereafter until discharge from the ICU or death. Specimens for cultures from other anatomical sites were ordered by the attending physician, as clinically indicated. For each patient and only at the onset of sepsis, blood specimens were simultaneously obtained for BG assay (from a peripheral venipuncture and/or an arterial line) and for culture (from a peripheral venipuncture and/or intravascular catheter). Blood cultures were processed using the automated BACTEC system (Becton Dickinson Diagnostic Instruments, Sparks, MD, USA).

### Definitions of IFIs and *Candida *colonization

Proven IFI was determined on the basis of the European Organization of the Research and Treatment of Cancer/Mycoses Study Group (EORTC/MSG) criteria [31], modified such that they could be applied with greater relevance to non-neutropenic adults [32], excluding BG detection as a microbiological criterion. Proven IC was defined by: i) histological evidence of yeast cells or hyphae or pseudohyphae from normally sterile site (that is, fluid obtained by drain), ii) blood culture positive for *Candida *species, or iii) positive culture result for sample from any other sterile site (excluding urine, sputum, bronchoalveolar lavage fluid, mucous membrane swabs, and specimens from skin sites) [32]. Candidemia was considered to be catheter-related if simultaneous quantitative cultures showed a ratio of ≥ 5:1 in colony-forming unit of blood samples obtained through the catheter and a peripheral vein. Patients with proven invasive mold infection had histological evidence of tissue invasion by filamentous fungi, the isolation of a mold from a normally sterile but clinically infected body site (excluding bronchoalveolar lavage fluid, a cranial sinus cavity specimen, and urine), or blood culture that yields a mold (for example, *Fusarium *species) in the context of a consistent infectious disease process [31].

*Candida *colonization was defined as positive culture for fungal species from any of the above mentioned surveillance sites. Colonization was considered, respectively, unifocal or multifocal when *Candida *species were isolated from one focus or simultaneously from various non-contiguous foci [26]. Candiduria was defined as the presence of at least 10^4 ^colony-forming units/mL of the same *Candida *species [26].

### CS and colonization index

The CS and colonization index were calculated when results of the patients' surveillance cultures, with the exception of blood cultures, were available. The CS for a cut-off value of 3 was as follows: total parenteral nutrition × 1, plus surgery × 1, plus multifocal *Candida *colonization × 1, plus severe sepsis × 2 [26]. The colonization index was calculated as the ratio of the number of culture-positive surveillance sites to the total number of sites cultured [24]. The cut-off points for discriminating between *Candida *species colonization and IC were ≥3 for the CS and ≥0.5 for the colonization index [24,25]. For each patient, maximum values recorded for CS and colonization index at or before the episode of IC were used in the analysis. In the absence of IC, the maximum of all observed values was used.

### BG assay

Patients' sera recovered from arterial and/or venous blood specimens (see above) were tested for BG (Fungitell^®^; Associates of Cape Cod Inc, Falmouth¸ MA, USA) as recommended by the manufacturer. The concentration of BG in each sample was automatically calculated using a calibration curve with standard solutions ranging from 31.25 to 500 pg/mL. The manufacturer's BG cut-off of 80 pg/mL was used. All samples were analyzed in triplicate and the mean was assigned as the final result for the specimen. Assay results were not reported to treating clinicians.

### Statistical analysis

Data were analyzed using STATA 10 (StataCorp LP, College Station, Texas, USA). Normally distributed continuous variables were reported as mean ± SD and compared using Student's *t *test. Medians with ranges were used to describe non-normally distributed continuous variables, and compared using the Mann-Whitney *U*-test. Categorical variables were reported as percentages and compared using the two-tailed χ^2 ^test or Fisher's exact test, as appropriate. The following parameters of diagnostic performance and their 95% confidence intervals (CIs) were calculated: sensitivity, specificity, positive and negative predictive value (PPV, NPV), and positive and negative likelihood ratios (PLR, NLR), and Cohen's kappa. The discriminatory powers for BG, CS, and colonization index were evaluated by their respective areas under the receiver operating characteristic (ROC) curves (AUCs). ROC AUC comparisons were performed using the method of Hanley and McNeil [33].

## Results

Of 377 patients with clinical signs of sepsis admitted to the ICU during the study period, 95 who fulfilled the inclusion criteria above specified were enrolled as participants (Figure 1). Characteristics of the 95 subjects (16 case patients in the IFI group and 79 patients in the group without evidence of IFI) are shown in Table 1. The majority of patients (74%) had at least one predisposing defined host (that is, use of corticosteroids or other immunosuppressants) and/or risk factors. Nonetheless, no patients with probable/possible IFI were identified on the basis of modified EORTC/MSG criteria.

**Table 1:** Demographics and clinical characteristics of studied groups

	Total (*n *= 95)	IFI^a^ (*n *= 16)	No IFI (*n *= 79)	*P*
Age, median years (range)	69 (18 to 93)	74 (53 to 82)	69 (18 to 93)	0.12
Male sex (%)	65 (68.4)	11 (68.8)	54 (68.4)	0.97
SAPS II, median (range)	47 (14 to 76)	49.5 (26 to 74)	46 (14 to 76)	0.18
SOFA, median (range)	6 (0 to 16)	5.5 (0 to 15)	6 (0 to 16)	0.33
ICU stay, median days (range)				
Before sepsis onset	7 (6 to 67)	8.5 (6 to 34)	7 (6 to 67)	0.95
Overall	20 (6 to 180)	28 (7 to 92)	20 (6 to 180)	0.24
ICU mortality (no.,%)	23 (24.2)	6 (37.5)	17 (21.5)	0.20
Diagnosis on ICU admission (no.,%) Medical Surgical Trauma	61 (64.2) 12 (12.6) 22 (23.2)	13 (81.3) 2 (12.5) 1 (6.2)	48 (60.8) 10 (12.7) 21 (26.6)	0.12 1 0.11
Multifocal *Candida *colonization (no.,%)	40 (42.1)	10 (62.5)	30 (37.9)	0.07
Abdominal surgery (no.,%)	11 (11.6)	2 (12.5)	9 (11.4)	1
Risk factors (no.,%)				
Mechanical ventilation	85 (89.5)	16 (100)	69 (87.3)	0.20
Central venous catheter	86 (90.5)	15 (93.7)	71 (89.9)	0.63
Broad to spectrum antibiotics	71 (74.7)	14 (87.5)	57 (72.2)	0.19
Total parenteral nutrition	10 (10.5)	7 (43.7)	3 (3.8)	< 0.001
Corticosteroids	8 (8.4)	3 (18.7)	5 (6.3)	0.13
Renal replacement therapy	12 (12.6)	5 (31.3)	7 (8.9)	0.03
Underlying diseases (no.,%)				
COPD	20 (21.1)	5 (31.2)	15 (19.9)	0.27
Solid transplants	1 (1.1)	1 (6.3)	0	0.17
HIV	1 (1.1)	1 (6.3)	0	0.17
Solid cancer	12 (12.6)	3 (18.7)	9 (11.4)	0.40
Hematological malignancy	1 (1.1)	1 (6.3)	0	0.17
Cirrhosis	1 (1.1)	1 (6.3)	0	0.17
Chronic renal failure	24 (25.3)	8 (50.0)	16 (20.3)	0.02
Diabetes	51 (53.7)	10 (62.5)	41 (51.9)	0.44
Clinical condition (no.,%)				
Severe sepsis	54 (56.8)	5 (31.2)	49 (62.0)	0.02
Septic shock	21 (22.1)	10 (62.5)	11 (13.9)	< 0.001
Pneumonia	29 (30.5)	5 (31.3)	24 (30.4)	1
Gram-positive bloodstream infection^b^	11 (11.5)	1 (6.2)	10 (12.6)	0.46
Gram-negative bloodstream infection^c^	7 (7.4)	0	7 (8.9)	0.21
Other bacterial infection^d^	15 (15.7)	3 (18.7)	12 (15.2)	0.72
No. of patients (%) with a:				
Positive BG result	20 (21.1)	15 (93.7)	5 (6.3)	< 0.001
CS value ≥3	21 (22.1)	12 (75.0)	9 (11.4)	< 0.001
Colonization index ≥0.5	33 (35.1)	9 (56.3)	24 (30.4)	0.04

BG, (1→3)-β-D-glucan; COPD; chronic obstructive pulmonary disease; CS, *Candida *Score; IFI, invasive fungal infection; SAPS II, Simplified Acute Physiology Score II; SOFA, Sequential Organ Failure Assessment; ^a ^Two patients developed pulmonary aspergillosis or fusariosis.

^b ^By coagulase-negative staphylococci (*n *= 5), *Staphylocccus aureus *(*n *= 3), *Enterococcus faecalis *(*n *= 2), or *Streptococcus mitis *(*n *= 1).

^c ^By *Klebsiella pneumoniae *(*n *= 2), *Pseudomonas aeruginosa *(*n *= 1), *Escherichia coli *(*n *= 1), *Providencia stuartii *(*n *= 1), *Enterobacter aerogenes *(*n *= 1), or *Proteus mirabilis *and *P. aeruginosa *(*n *= 1).

^d ^Include urinary tract, wound, or fluid infections.

The overall prevalence of IFI was 16.8%. Proven cases included 2 pulmonary mold infections (1 with *Aspergillus fumigatus *and 1 with *Fusarium solani*) and 14 *Candida *infections, of which 13 candidemia (10 with *C. albicans*, 1 with *C. glabrata*, 1 with *C. parapsilosis*, and 1 with *C. tropicalis*) and 1 mediastinitis (with *C. albicans*). Eighteen patients had documented bacterial bloodstream infections (1 with and 17 without concomitant IFI).

Thirteen of 95 patients (only 1 of the IFI group) were receiving empirical antifungal therapy (7 with echinocandin and 6 with fluconazole) two to four days before testing.

### Blood sampling for BG serum measurement

A total of 130 serum samples were tested with the BG assay. For the first enrolled 35 patients, BG was measured in 70 paired sera from their respective venous and arterial blood specimens, which were collected concomitantly in order to assess the equivalence of measuring BG in blood samples obtained from peripheral venipuncture or from arterial catheters. This is also in consideration that the pre-analytical environmental contamination by BG of the tested blood is generally considered a major limitation of this assay.

**Figure 2:**
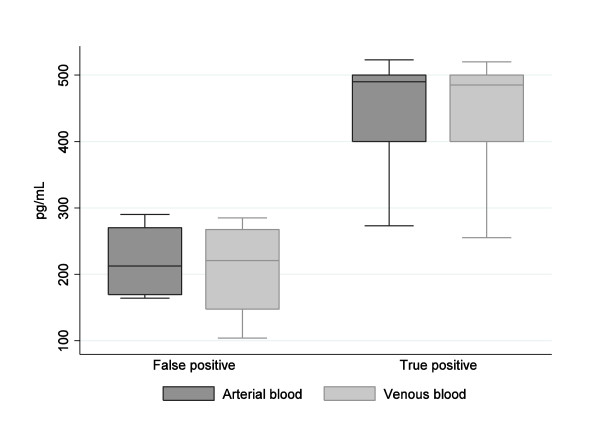
**(BG results of sera from two blood sampling sites for subjects with or without IC**.

Using the predefined cut-off value of 80 pg/mL, 10 of 35 patients had arterial and venous sera that tested as positive for BG. For the remaining 25 patients, both the serum specimens tested as negative for BG (range, 10 to 65 pg/mL). The BG levels obtained in the arterial and venous specimens were not different: 355.5 ± 140.9 and 347.5 ± 148.9 pg/mL, respectively (*P *= 0.24). Figure 2 shows the overall test results for the 10 BG-positive patients according to IC diagnosis. The BG level in the true BG-positive sera (464 ± 81 pg/mL) was significantly higher than in the false BG-positive sera (217 ± 115 pg/mL) (*P *< 0.001). Since the BG assay worked well also with the serum retrieved from non-venous blood sampling, only arterial blood specimens were subsequently used for BG determination in the other 60 study participants.

Overall, sera from 95 patients were evaluated. Patients with IFI were compared to patients who had no identified IFI according to the ICU-adapted EORTC/MSG criteria (Table 1). Fifteen of 16 IFI patients yielded BG-positive results. Among the patients who had no IFI, only 5 of 79 patients tested had BG-positive results. The sensitivity, specificity, PPV, and NPV of BG testing for the diagnosis of IFI in all 95 patients were 93.7%, 93.6%, 75.0%, and 98.6%, respectively.

### IC according to BG assay, colonization index, and CS

Fourteen of the 95 patients with BG serum level determinations (Table 1) had an ongoing IC. In 13 the BG was positive and in 1 was negative. The features of these patients are summarized in Table 2. The mean BG level was 436.42 ± 132.13 pg/mL, whereas the median of the BG levels for the 14 patients was 500 (range 27 to ≥500) pg/mL. The patient with a negative BG test had received, four days before the serum sampling, antifungal treatment with caspofungin, an antifungal drug that interferes with BG synthesis. In all 13 patients, a positive BG assay result was obtained 24 to 72 hrs before a positive bloodstream culture result.

**Table 2:** Fourteen patients with proven invasive *Candida *infection and their serum BG measurements before diagnosis

**No**.	SAPS II	Underlying conditions	Clinical syndrome/admission diagnosis	Type/source of infection	BG level (pg/mL)^a^	Antifungal treatment	Outcome	LOS ICU (days)
8	35	Steroid therapy, solid cancer	Respiratory failure, severe sepsis	Candidemia/unknown	≥500	Caspofungin	Alive	30
15	48	Diabetes, obesity, chronic renal failure	Septic shock	Candidemia/UTI	≥500	Anidulafungin	Dead	7
18	69	Chronic renal failure, diabetes	Respiratory failure, septic shock	Mediastinitis/Unknown	≥500	Anidulafungin	Dead	12
21	64	COPD	Septic shock	Candidemia/unknown	≥500	Anidulafungin	Alive	8
29	61	Diabetes, chronic heart failure	Respiratory failure, severe sepsis	Candidemia/UTI	480	Anidulafungin	Dead	92
32	67	Chronic heart failure, solid cancer	Respiratory failure	Candidemia/CVC	400	Fluconazole	Alive	26
35	45	Hematological malignancy, diabetes	Septic shock	Candidemia/unknown	255	Anidulafungin	Dead	7
51	65	AIDS, cirrhosis	Septic shock, ALI	Candidemia/CVC	322	Caspofungin	Dead	7
57^b^	59	Obesity, solid cancer	Respiratory failure, septic shock	Candidemia/CVC	27	Caspofungin	Dead	14
60	39	Renal transplant, steroid therapy	Trauma, severe sepsis	Candidemia/CVC	≥500	Caspofungin	Alive	35
74	51	Intestinal occlusion, chronic renal failure	Respiratory failure, severe sepsis	Candidemia/unknown	≥500	Anidulafingin	Alive	65
79	26	COPD, chronic hearth failure, diabetes	Septic shock, respiratory failure	Candidemia/CVC	≥500	Anidulafungin	Alive	45
87	41	Chronic hearth failure, COPD, diabetes, abdominal surgery	Severe sepsis, respiratory failure	Candidemia/UTI	≥500	Caspofungin	Alive	45
89	30	Chronic hearth failure, abdominal surgery, chronic renal failure	Septic shock	Candidemia/UTI	≥500	Caspofungin	Alive	50

^a ^Values of ≥500 pg/mL were converted to the 500-pg/mL ones for statistical analysis.

^b ^This patient was empirically treated with caspofungin before BG testing.

ALI, acute lung injury; COPD; chronic obstructive pulmonary disease; CVC, central venous catheter; LOS, length of stay; SAPS II, Simplified Acute Physiology Score II; UTI, urinary tract infection

A number of surveillance specimens from the 95 patients were screened for the presence of *Candida *species. A colonization index ≥0.5 was found in 30.4% (24/79) of no-IFI patients and in 56.3% (9/16) of patients with IFI (*P *= 0.04) (Table 1). IC according to the colonization index occurred in 7 of 33 patients.

As shown in Table 1 the percentage of patients with severe sepsis was significantly higher in the no-IFI group (*P *= 0.02), whereas septic shock was significantly higher in patients with IFI (*P *< 0.001). Total parenteral nutrition was also significantly higher in the group of IFI (*P *< 0.001). Ten of 21 patients with a CS ≥3 had an ongoing IC.

**Figure 3:**
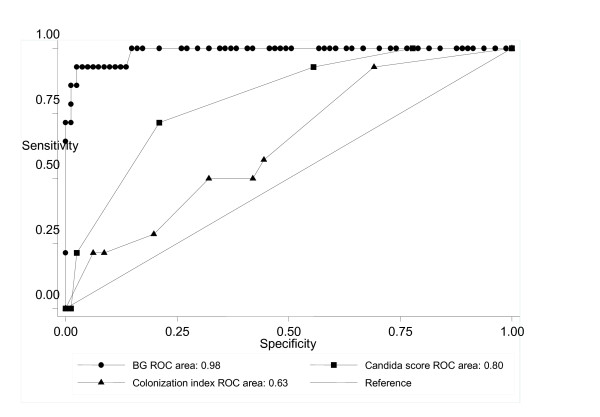
**ROC AUC curves of BG, CS, and colonization index for proven IC cases**. [The AUC of BG was significantly higher than those of CS (*P *< 0.001) and colonization index (*P *< 0.001), please edit this sentence as a footnote].

Diagnostic test indices for IC are presented in Table 3. BG had better results when compared with CS and colonization index in all studied variables. In particular, the BG test gave a significantly higher number of positive results with a sensitivity of 92.9% versus 85.7% of CS and 64.3% of the colonization index. The specificities of BG, CS, and colonization index were also dissimilar (93.7%, 88.6%, and 69.7%, respectively). The areas under the ROC curves for BG, CS, and colonization index were 0.98 (95% CI 0.92 to 1.00), 0.80 (95% CI 0.69 to 0.92), and 0.63 (95% CI 0.57 to 0.79) (Figure 3). The agreement between BG assay and CS was moderate (kappa = 0.53) and that between BG assay and colonization index was poor (kappa = 0.10).

**Table 3:** Performances of (1→3)-β-D-glucan assay (BG), *Candida *score (CS), and colonization index for detection of invasive candidiasis in 95 patients

	Sensitivity (%) (95% CI)	Specificity (%) (95% CI)	PPV (%) (95% CI)	NPV (%) (95% CI)	PLR (%) (95% CI)	NLR (%) (95% CI)
BG cut-off value, 80 pg/mL	92.9 (66.1 to 99.8)	93.7 (85.8 to 97.9)	72.2 (46.5 to 90.3)	98.7 (92.8 to 99.9)	14.74 (4.65 to 47.52)	0.07 (0.02 to 0.39)
CS ≥3	85.7 (57.2 to 98.2)	88.6 (79.5 to 94.7)	57.1 (34.0 to 78.2)	97.2 (90.3 to 99.7)	7.51 (2.79 to 18.29)	0.16 (0.02 to 0.54)
Colonization index ≥0.5	64.3 (35.1 to 87.2)	69.6 (58.2 to 79.5)	27.3 (13.3 to 45.5)	91.7 (81.6 to 97.2)	2.12 (0.84 to 4.25)	0.51 (0.16 to 1.11)

The combination of a positive BG result and a CS value ≥3 increased the sensitivity (100% (95% CI, 76.8% to 100%)) and NPV (100% (95% CI, 94.6% to 100%)) for diagnosis of IC, compared to 92.9% and 97.2% for the BG test alone, respectively. Conversely, the specificity (83.5% (95% CI 73.5% to 90.9%)) and PPV (51.8% (95% CI, 31.9% to 71.3%)) based on combined tests was lower than those of the BG test (93.7% and 72.2%, respectively).

Among the 5 patients with false-positive BG tests, 4 were treated with β-lactams and 1 was exposed to surgical gauze, whereas 36 of the BG-negative patients were receiving β-lactam antibiotic therapy and 4 underwent a surgical gauze packing before BG testing.

## Discussion

Using a single sample of patients obtained at the onset of the septic syndrome in our evaluation, which included 16 subjects with IFI and 79 with no IFI, a BG level ≥80 pg/mL had sensitivity and specificity of 93.7% and 93.6% for confirmed IFI, respectively. With respect to prior studies [15,34], overall BG assay sensitivity was higher in our cohort, likely because of lack of subjects in the EORTC/MSG categories with inherent diagnostic uncertainty (possible or probable IFI) and/or in whom no active IFI occurred at the time of sampling. Accordingly, BG sensitivity for patients with proven IC was 92.9%, whereas all but five episodes of non-fungal infections had BG concentrations higher than the cut-off value (specificity 91.4%).

In a multicenter evaluation of the Fungitell kit conducted in the United States by Ostrosky-Zeichner *et al. *[35], the sensitivity of the assay for patients with proven IC (*n *= 107; cut-off, 80 pg/mL) was 77.6% based on a single sample obtained from the patient within 72 hrs after entry into the study. A single-sample strategy was also adopted in studies that, similarly to ours, involved patients who had or developed systemic *Candida *infections (for review, see Karageorgopoulos *et al. *[36] and the references therein), in which BG assay sensitivity for proven or probable IC ranged from 85% [19] to 92% [37]. Although obtaining multiple samples increased specificity and PPV of the BG assay for subjects with acute myeloid leukemia or myelodysplastic syndrome [16], this may be not the case for ICU patients, for whom it could be important to explore the performance of the assay with a single-sample protocol. This protocol was believed to be potentially closer to the way in which the assay could be used in ICU clinical settings [35].

Our BG sampling strategy involved the use of a blood specimen drawn from an arterial line. In the ICU setting, arterial catheters allow continuous real-time blood pressure monitoring and reduce the need for repeated punctures for blood gas analysis. Therefore, it was reasonable to assess whether this blood source could be exploited for microbiological tests, instead of a central venous catheter (nine of our patients did not harbor this device). This was, also, in view of a recent study showing no significant statistical difference in rates of contamination between venous and arterial sites of blood draw [38], at least for patients not particularly vulnerable to fungal colonization/infection, such as burn patients. To date, this is the first evaluation enabling use of the Fungitell kit with sera retrieved from arterial blood specimens, and, in our opinion, information that measurements obtained from either arterial or venous blood samples are equivalent. It could represent a new and very useful practical aspect.

The use of certain intravenous antimicrobials (for example, amoxicillin-clavulanic acid) or gauze packing of serosal surfaces may be a source of false-positive BG measurements [39]. We detected a high level of BG in five patients without evidence of fungal infection, one of whom was undergoing extensive intra-abdominal gauze exposure following surgery and four were receiving antimicrobial treatment with β-lactams. However, considering that 4 of the patients with true-negative BG results were exposed to surgical gauze and 36 were treated with β-lactam antibiotics, it is unlikely that a casual relationship between the presence of BG in the blood specimens and medical interventions exists in our patients. Despite the wide use of intravenous albumin or immunoglobulins and/or hemodialysis in ICU patients, such medical treatments did not lead to a false-positive BG elevation in our patients. Some studies also reported a high number of false positives in patients with bacteremia, especially that caused by Gram positive organisms [40,41]. In one study comparing patients with blood cultures positive for yeast (proven or probable IFI) versus bacteremic patients, this appeared to reduce the specificity of the test and to place its PPV at 52%, but given the nature of the samples obtained the authors admitted that BG contamination may have occurred [40]. In another study analyzing a single specimen in 46 subjects from ICUs, Digby *et al. *[41] observed that serum BG levels were elevated on average in all patients with either fungal or bacterial infection, even though hemodialysis as a source of false-positivity may be a factor in these observations. By contrast, no cross-reactivity of the BG assay in patients experiencing bacteremias was found in our study and in the prospective validation assays published to date [15-17,34].

Multisite colonization is a predictor for candidemia in the ICU, especially in combination with other risk factors reflecting severe disease [4,42]. Thus, IC is highly improbable if a *Candida-*colonized critically ill patient has a CS < 3 [26]. Colonization by fungi was seen to not raise the concentration of BG [16,34], while BG may reach the circulation through damaged intestinal or respiratory mucosa and causes positive test results. In this case, BG could be the marker of the gut barrier invasion prior to infection. Of interest, studying a patient population different from ours but with similar risk profiles, Senn *et al. *[17] showed that all of the false-positive BG assay results (4%) were observed in patients with gastrointestinal fungal colonization and/or mucositis who received empirical therapy, suggesting an occult IC at an early stage. Few studies have performed direct comparisons of the BG assay, CS, and colonization index for identifying patients at risk of development of IC [26]. In our study, we found that 10 of 13 BG positive patients with proven IC (true positive) had a CS ≥3 and 7 a colonization index ≥0.5, and these findings, on the one hand, reinforce the discriminatory power of BG testing between patients with true IC and those without IC and, on the other hand, suggest its use to make diagnosis more quickly. However, the not too high concordance observed warrants additional investigations whether the three tests can be used as complements to each other, although an analysis of diagnostic performance combining BG assay and CS showed that combined use of the two tests enhanced the sensitivity and NPV for the diagnosis of IC.

Aside from the low frequency of cases of proven IC, the experimental design of our study, giving an estimation of the prevalence of IFI in the target population, allowed an assessment of PPV and NPV by use of BG testing. With a prevalence of fungal infection of 16.8%, the PPV for the BG test was 72.2%, in spite of a very high NPV (approximately 99%). Therefore, a strong diagnostic benefit of BG testing lies in excluding IFI (and IC), according to that reported earlier [20,35,43], while an unresolved question about the BG testing remains the knowledge of factors that could increase BG levels for reasons other than IFI. Nevertheless, the small number of subjects studied, leading to broad CI intervals, should lower the strength of our observations. Additionally, the pre-test probability of IC in our study population was high given that 74% of the patients presented with risk factors for IFI, thus possibly limiting the generalizability of our data to other patients. However, a strict but effective patient selection strategy such as that adopted in this study, if combined with a single-test approach, may have a major financial impact by reducing the laboratory costs associated with the use of this expensive biomarker.

We are aware of the potential limits of BG assay for detecting fungal infection that would yet balance key deficiencies in the current culture-based methods, such as poor sensitivity and delayed turnaround time. A similar scenario is offered by newer PCR techniques (for example, SeptiFast; Roche Molecular Systems, Mannheim, Germany), though their application to detect and identify fungal pathogens more rapidly than conventional culture might revolutionize the diagnosis and the management of sepsis [44]. However, provided an appropriate laboratory logistics, BG assay results can be obtained within a few hours, and then more rapidly than those of PCR techniques, which remain extremely complex and expensive for a routine microbiology laboratory.

## Conclusions

The BG assay, based on arterial blood sampling, appears to be useful as a single-point assay for ICU patients with suspicion of *Candida *sepsis. In addition, a combination of CS and BG measurement in this setting may improve the diagnostic performance and the efficiency of management. However, this strategy deserves further study to assess its true value in early detecting ICs and thus reducing the increased mortality [3] and cost of ICU stay [45] caused by *Candida *infections.

## Key messages

• A timely diagnosis of IC in medical ICU patients with signs of sepsis is essential to reduce the morbidity and mortality associated with *Candida *infection.

• The culture-independent serum BG assay may allow early diagnosis of IC, even when one patient sample is tested at the onset of the septic syndrome.

• The NPV for BG testing in our study population is nearly 99%, suggesting that a strong diagnostic benefit of this assay lies in excluding IC.

• In all proven-IC cases, a positive BG result was obtained 24 to 72 hrs before a positive bloodstream culture result, thus overcoming the delayed turnaround time of conventional diagnostic methods.

• A combined use of BG and CS may improve the diagnostic performance in ICU patients at risk for *Candida *sepsis, but additional investigations are needed.

## Abbreviations

ALI: acute lung injury; AUC: area under the curve; BG: (1→3)-β-D-glucan; CI: confidence interval; COPD: chronic obstructive pulmonary disease; CS: *Candida *score; CVC: central venous catheter; EORTC/MSG: European Organization of the Research and Treatment of Cancer/Mycoses Study Group; IC: invasive candidiasis; IFI: invasive fungal infection; NLR: negative likelihood ratio; NPV: negative predictive value; PLR: positive likelihood ratio; PPV: positive predictive value; SAPS II: Simplified Acute Physiology Score II; SOFA: Sequential Organ Failure Assessment; ROC: receiver operating characteristic; UTI: urinary tract infection

## Competing interests

The authors declare that they have no competing interests.

## Authors' contributions

BP, GF, MS and MA participated in the design and coordination of the study. RT participated in the mycological analysis. MAP, GB, and RM were attending physicians of the patients in the ICU. GDP collected the primary datasets. MT performed data analysis and helped draft the manuscript. BP performed background literature review and wrote the manuscript. All authors read and approved the final manuscript.
